# Optimization of Metagenomic Library Construction for Influenza A Virus and SARS-CoV-2: Systematic Comparison of rRNA Depletion Strategies and Fragmentation Orders

**DOI:** 10.3390/diagnostics16132065

**Published:** 2026-07-01

**Authors:** Yi Sun, Feng Wang, Lingfeng Mao, Wenjun Lu, Hao Wu, Haiyan Mao, Yanjun Zhang

**Affiliations:** 1 Zhejiang Key Laboratory of Public Health Detection and Pathogenesis Research, Department of Microbiology, Zhejiang Provincial Center for Disease Control and Prevention, Hangzhou 310051, China; ysun@cdc.zj.cn (Y.S.); fwang@cdc.zj.cn (F.W.); 2 Hangzhou Baiyi Technology Co., Ltd., Hangzhou 310051, China; mao_lingfeng@foxmail.com (L.M.); luwenjun@baiyi-tech.cn (W.L.); wuhao@baiyi-tech.cn (H.W.)

**Keywords:** RNA virus, metagenomic library construction, rRNA depletion, fragmental strategy, genomic coverage, host read contamination

## Abstract

**Background/Objectives**: RNA virus metagenomic sequencing is a core technology for emerging infectious disease prevention and control, as well as for rapid pathogen identification. However, two major bottlenecks hinder its clinical application: the low fraction of informative sequencing reads caused by host rRNA contamination, and insufficient viral genome coverage. This study aimed to optimize the experimental parameters of RNA virus metagenomic sequencing, address the above bottlenecks, and establish a standardized workflow. **Methods**: Forty-five clinically positive samples (20 influenza virus-positive; 25 SARS-CoV-2-positive) were investigated in three parallel comparative experiments: rRNA depletion versus no depletion; probe-mediated RNase H digestion versus rRNA blocking; and two fragmentation timing strategies (fragmentation before versus after reverse transcription). Sequencing was performed on the GeneMind platform, and key performance metrics were systematically analyzed. **Results**: Following rRNA depletion, the host sequence proportion in the influenza virus and SARS-CoV-2 samples decreased from 39.5 to 90.5% to 3.6 to 32.2%, while the 10× genomic coverage increased from 0 to 99.4% to 98.1 to 100.0%. The proportion of host sequences captured by probe capture depletion (0.3–16.2%) was significantly (*p* < 0.05) lower than that captured by rRNA blocking module (14.3–92.3%). No significant differences were observed in the 10× genomic coverage (96.5–100.0%) or the fraction of effective viral reads between the two fragmentation strategies (*p* > 0.05). rRNA depletion is key to improving library quality, with post-capture probe digestion being optimal. **Conclusions**: The suggested optimization process will enhance sequencing efficiency and support the standardization of clinical RNA virus identification.

## 1. Introduction

Respiratory RNA viruses represent a major global public health threat. They are characterized by high transmissibility, rapid genetic evolution, and the ability to cause sudden large-scale outbreaks [1]. Among them, influenza A virus and SARS-CoV-2 are the most well-characterized high-risk representatives and have caused the most devastating pandemics in recent history, inflicting enormous morbidity, mortality, and socioeconomic burden worldwide [2]. Compared with intestinal pathogens, respiratory pathogens such as these have received greater attention from governments and international organizations and have become the focus of extensive research into model development, public health emergency preparedness, and forward-looking academic discussions [3].

Historically, disease control and prevention and laboratory and clinical diagnosis of infectious diseases have relied largely on microscopic examination and culture in appropriate media or cell lines. The advent of molecular biological techniques and sensitive RNA/DNA detection-by-amplification methods has dramatically changed clinical practice for infectious diseases. However, such assays require prior knowledge of the causative pathogen, and not all molecular assays are readily available in clinical settings for every suspected pathogen. By contrast, metagenomic next-generation sequencing (mNGS) is a bias-free method that retains the key advantages of molecular tests and requires no information on the etiology of the disease. This method allows for the detection of a wide range of microbes (viruses, bacteria, fungi, and parasites) present in a sample in a single assay [4]. Beyond clinical diagnosis, mNGS has demonstrated great potential for novel pathogen discovery, as evidenced by its pivotal role in the recent outbreak of SARS-CoV-2-associated pneumonia [5,6]. Thus, mNGS has widespread microbiological applications, including infectious disease diagnosis in clinical laboratories, pathogen identification for acute and chronic illnesses of unknown origin, and outbreak surveillance on a global scale [5].

Although mNGS has significant advantages, there are still several technical and regulatory obstacles to its widespread clinical application. The most obvious limitation is that the entire process usually takes several days and involves a series of wet and dry laboratory activities, and its reliability requires strict validation. In particular, wet laboratory procedures typically involve extracting small amounts of nucleic acids and converting them into high-quality sequencing libraries. Three critical issues remain: the necessity of rRNA depletion; the variable performance of different rRNA depletion techniques; and the impact of fragmentation order on library quality. These aspects require further additional experimental data and analytical validation, especially for metagenomic sequencing-based respiratory virus detection. In clinical samples such as throat swabs, host rRNA accounts for 80–90% of total RNA, leading to a low proportion of effective viral sequences in sequencing data, incomplete genome coverage, increased sequencing costs, and prolonged analytical cycles [7]. Two main categories of rRNA depletion techniques have been developed to address this core bottleneck: (1) pull-out methods that physically separate rRNA from other RNA species; (2) enzymatic degradation methods that selectively digest rRNA using RNase H after hybridization with sequence-specific DNA probes. Both approaches use oligonucleotide probes complementary to human cytoplasmic rRNAs (5S, 5.8S, 18S, 28S) and mitochondrial rRNAs (12S, 16S), which are often premixed for convenient sample processing and can also be adapted to deplete other high-abundance transcripts such as globin mRNA [7,8,9]. Commercial implementations of RNase H-based methods include NEBnext RNA depletion (New England Biolabs, Hitchin, UK) and RiboErase (Kapa Biosystems, Cape Town, South Africa), which degrade the resulting DNA-RNA hybrids to remove rRNA from the sample. However, direct head-to-head comparative studies evaluating the performance of these different rRNA depletion strategies specifically for RNA virus metagenomics remain rare. Additionally, the choice of fragmentation strategy for RNA virus library construction (i.e., fragmentation followed by reverse transcription vs. reverse transcription followed by fragmentation) is still largely empirical, lacking performance evaluation in large clinical sample sizes. Therefore, optimizing current methodologies and translating them into a robust, standardized protocol is essential for reliable RNA virus metagenomic sequencing in clinical practice, particularly for challenging low-viral-load samples.

This study focused on two representative RNA viruses—influenza A (H1N1) virus and SARS-CoV-2—detected in clinical throat swab samples. Three sets of comparative experiments were designed: (1) rRNA depletion versus no depletion; (2) probe-mediated RNase H digestion versus rRNA blocking; and (3) fragmentation before reverse transcription versus fragmentation after reverse transcription. We systematically compared key performance metrics including target reads per million, 10× genomic coverage, and host sequence proportion, and established a standardized library construction workflow for RNA virus metagenomics. Our findings provide an experimental basis for optimizing key technical parameters of RNA virus metagenomic library construction, support the standardization of identification of RNA viruses including SARS-CoV-2 and influenza virus, and enable medical and public health authorities to achieve a rapid response and accurate pathogen identification during emerging infectious disease outbreaks.

## 2. Materials and Methods

### 2.1. Experimental Materials

The samples for this study were clinical throat swab samples provided by the Zhejiang Provincial Center for Disease Control and Prevention (45 cases), including 20 influenza virus and 25 SARS-CoV-2 samples, which were verified by RT-PCR (Yeasen, Shanghai, China) as positive for FluA (H1N1) pdm09 and the ORF1ab/N gene of SARS-CoV-2. These 45 samples were randomly selected from a larger cohort of 332 well-characterized clinical throat swab samples using a computer-generated random number sequence. The study was approved by the institutional Ethics Committee (Ethical number: 2024-068-01). All samples were stored at −80 °C and rapidly thawed before the study. RNA was extracted using the RNeasyMiniKit (50) (74104, QIAGEN, Düsseldorf, Germany).

### 2.2. Experimental Design

**Figure 1:**
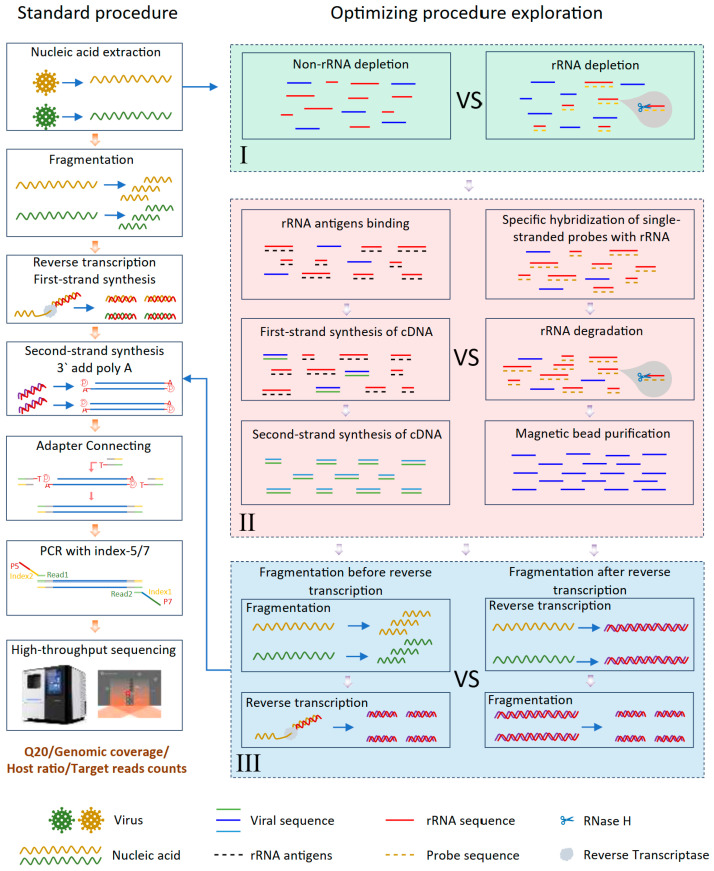
Workflow schematic of respiratory RNA virus metagenomic sequencing and procedure optimization. A standard workflow without rRNA depletion and three sets of optimized strategies are illustrated: non-rRNA depletion or rRNA depletion methods in module I; two host rRNA depletion methods in module II (probe-mediated RNase H degradation and rRNA antigen-binding); and two fragmentation timing strategies in module III. Key sequencing metrics including host ratio, target reads per million, Q20, and genomic coverage are evaluated for cross-protocol comparison.

A paired experimental design was employed in this study, where all 45 samples were used in all three of the comparative experiments described below. Each RNA extract was divided into equal aliquots for parallel processing under different experimental conditions, ensuring complete sample overlap across all experiments and eliminating variability introduced by different RNA isolation batches, to minimize potential batch effects. All library preparation and sequencing steps were performed by the same technician in a single continuous batch within a 2-week period, samples were processed in a randomized order during library preparation, and negative controls were included at every step of the workflow to monitor for contamination. The study was divided into three parts. The first part compared the rRNA depletion and non-rRNA depletion groups. rRNA was depleted from the samples using a Pathogen Metagenomic RNA-seq Library Prep Kit (BK-RLA024, Baiyi, Hangzhou, China), which incorporates a probe-mediated RNase H enzymatic degradation method for rRNA depletion. Reverse transcription and library construction were performed using the rRNA depletion group. The direct enzymatic depletion step was omitted for library construction using a Pathogen Metagenomic RNA-seq Library Prep Kit (BK-RLA024, Baiyi, Hangzhou, China) for the non-rRNA depletion group. The second part compared the two rRNA depletion methods: probe-mediated RNase H degradation (BK-RLA024, Baiyi, Hangzhou, China), hereinafter referred to as rRNA degradation, and probe-based rRNA blocking (Fast Select rRNA Kit (Human), N460, Vazyme, Nanjing, China), hereinafter referred to as rRNA blocking. The samples in both parts of the experiment were obtained as cDNA through reverse transcription, and libraries were constructed by using a Pathogen Metagenomic RNA-seq Library Prep Kit (BK-RLA024, Baiyi, Hangzhou, China). The third part compared the two fragmentation methods. After rRNA degradation, group one underwent fragmentation and reverse transcription, followed by library construction and sequencing. Group two underwent reverse transcription and fragmentation before library construction and sequencing. All libraries were sequenced on the GeneMind FASTAseq 300 platform (500 M chip, GeneMind, Shenzhen GeneMind Biotech Co., Ltd., Shenzhen, China), which employs a sequencing-by-synthesis chemistry similar to Illumina platforms and generates 150 bp paired-end reads. Compared with the Illumina NovaSeq 6000, the FASTAseq 300 exhibits comparable substitution error rates (~0.1%) and slightly higher indel error rates (~0.02% vs. ~0.01% for Illumina), which is well within the acceptable range for viral metagenomic sequencing. All samples were sequenced on the same platform in a single batch to eliminate any platform-specific bias from our comparative analyses. The entire process is shown in Figure 1.

In most RNA sequencing (RNA-seq) studies, it is desirable to eliminate rRNAs so that as many reads as possible come from mRNAs. To enrich the mRNA in RNA-seq samples, a common strategy involves depletion of rRNAs by subtractive hybridization [10]. The Pathogen Metagenomic RNA-seq Library Prep Kit (BK-RLA024, Baiyi, Hangzhou, China) employs a probe-mediated RNase H degradation approach. After specific rRNA probes hybridize with the rRNA of the target species, RNase H and DNase I are used for stepwise enzymatic digestion to remove rRNA. This method involves a relatively complex workflow but offers high specificity and a high recovery rate for target RNA. It is compatible with 0.1–1 μg of intact RNA input and is primarily applied for the detection of unknown RNA viruses and the construction of metagenomic sequencing libraries. By contrast, the FastSelect rRNA Kit (Vazyme, Nanjing, China) uses an antigen-binding-mediated rRNA depletion method. It only requires mixing rRNA antigen-containing reagents with human total RNA samples (1 ng–1 μg total RNA input, compatible with both intact and degraded RNA) followed by a single incubation step. This protocol features a simple, time-saving workflow and requires fewer reagent components. It is primarily designed for human transcriptome (mRNA/ncRNA) profiling and is compatible with a wide range of library preparation kits.

### 2.3. Metagenomic Sequencing Data Processing

Metagenomic sequencing was performed using the GeneMind (Shenzhen, China) short-read strategy, yielding 150 bp paired-end reads with an overall depth of at least 100× per sample. All downstream analyses, including read filtering, genome assembly, sequence alignment, gene prediction, and functional annotation were conducted on the pathogenic microorganism bioinformatics analysis platform (Baiyi, Hangzhou, China). GeneMind raw reads were filtered with fastp v0.23.2 [11] to obtain clean data, removing read pairs in which either mate contained >10% ambiguous bases (N) or >50% bases with Phred quality score ≤ 5. Filtered short reads were assembled using bwa based on reference genomes. Sequencing depth was estimated by mapping GeneMind reads to the final assemblies with BWA v0.7.17 [12], followed by coverage calculation using the samtools v1.15.1 [13] depth command using a 2000 bp sliding window. Gene prediction and functional annotation of influenza A virus were performed using gfflu v0.02 (https://github.com/CFIA-NCFAD/gfflu, accessed on 5 January 2026). Host-derived reads were identified using Kraken2 v2.17.1 [14], and microbial abundance profiles were further corrected using Bracken v3.1 [15] within the broad-spectrum microbial analysis module of the platform.

### 2.4. Statistical Analysis

Key performance metrics for metagenomic sequencing data analysis, including data volume (Gb), total sequence number, number of target virus sequences, 10× coverage, number and proportion of host sequences, and proportion of valid data, all underwent statistical analysis. Statistical analyses were performed using GraphPad Prism v10 (Version 2025) and R v4.5.0 [16]. Data were summarized as means with standard deviations or medians with interquartile ranges, as appropriate. Group differences were assessed by paired *t*-test. When multiple comparisons were involved, *p* values were adjusted using the Benjamini–Hochberg false discovery rate procedure, and adjusted *p* < 0.05 was considered statistically significant. All statistical comparisons were performed using two-sided paired *t*-tests. The Bland–Altman method, a statistical approach for evaluating the agreement between two measurement methods, was performed by calculating the mean difference (bias) and standard deviation of differences to establish 95% limits of agreement and visualizing the relationship between measurement differences and their means in a scatter plot [17].

## 3. Results

### 3.1. rRNA Depletion Reduces the Proportion of Host Sequences and Improves Library Construction

**Figure 2:**
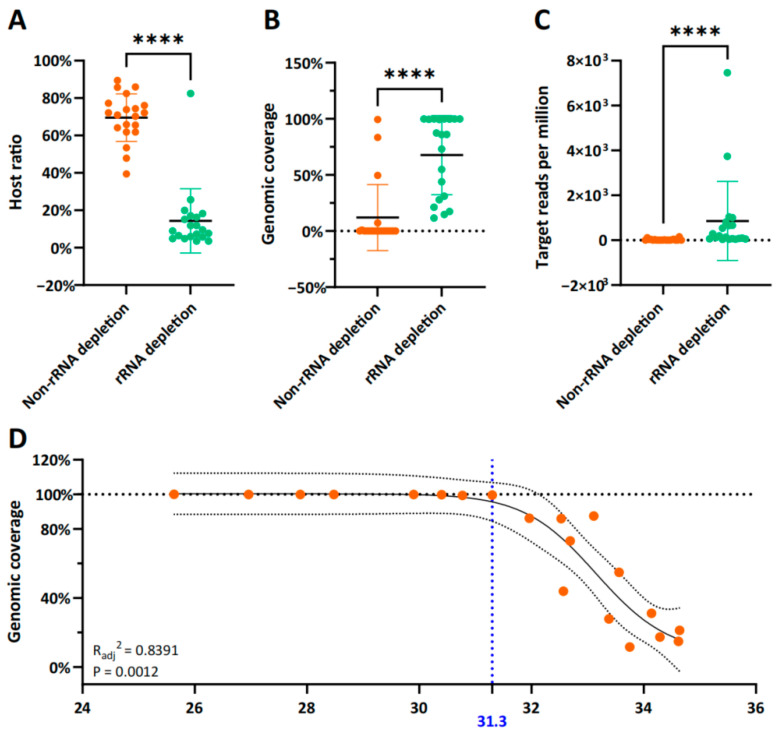
Comparison of influenza virus metagenomic sequencing using the rRNA depletion and non-rRNA depletion processing methods. (**A**–**C**) Host ratio, genomic coverage, and target reads per million of the two rRNA processing methods. (**D**) Fitted graph of the Ct values and genomic coverage of the RNA depletion samples (influenza virus, Ct range: 25.5–34.7). ****, *p* < 0.0001.

We performed metagenomic sequencing on 20 clinical influenza A virus-positive samples, with paired rRNA-depletion and no-depletion treatment groups, and evaluated the key performance metrics (Figure 2). The host ratio in the group without rRNA depletion ranged from 61.87 to 89.40%, while in the group with rRNA depletion, the ratio dropped to 3.6–25.7%, which was significantly lower (*p* < 0.001, paired *t*-test) than that in the group without rRNA depletion (Figure 2A). The 10× genomic coverage of the rRNA depletion group was 99.4–100.0%, which was significantly higher than that of the non-rRNA-depletion group (0–49.62%) (Figure 2B). The target reads per million of the rRNA depletion group were also significantly (*p* < 0.001, paired *t*-test) higher than that of the non-rRNA depletion group (Figure 2C). We performed a nonlinear fit of the amplification Ct values and the influenza genomic coverage of the rRNA depletion group (sample Ct range: 25.5–34.7). The correlation coefficient between the Ct value and the genome coverage reached 0.8391. When the Ct value was <31.3, the whole genome could be covered (10× coverage ≥ 99%) after rRNA depletion (Figure 2D). These results indicate that for RNA viruses such as the influenza virus and SARS-CoV-2, depletion of rRNA can reduce the host sequence and increase the genome coverage and proportion of valid data.

**Figure 3:**
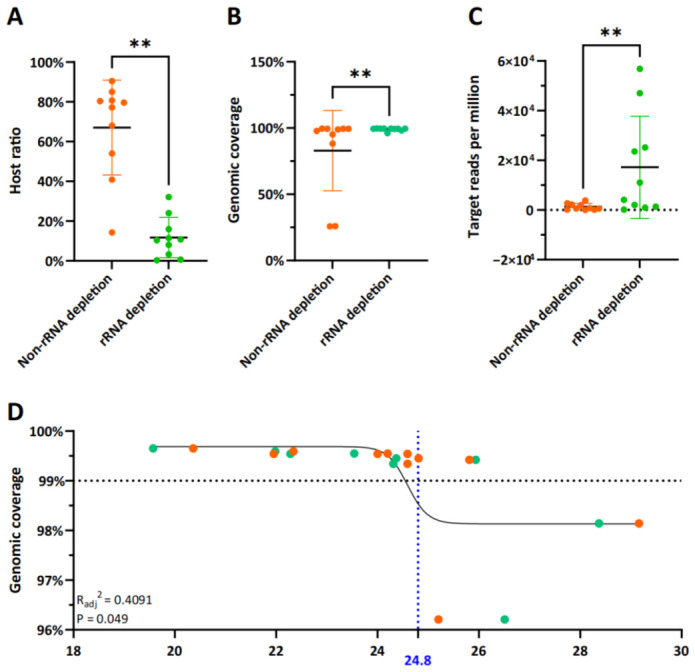
Comparison of SARS-CoV-2 metagenomic sequencing using rRNA depletion and non-rRNA depletion processing methods. (**A**–**C**) Host ratio, genomic coverage and target reads per million of the two rRNA processing methods. (**D**) Fitted graph of Ct values and genomic coverage of the SARS-CoV-2 samples (SARS-CoV-2, Ct range: 19.5–29.2). Color: orange circle = non-rRNA depletion samples, green circle = rRNA depletion samples. The blue dot line means the whole genome could be covered (10× coverage ≥ 99%) with Ct value < 24.8. **, *p* < 0.01.

We compared the core indicators of 10 RNA-based whole-genome sequencing of SARS-CoV-2 (Figure 3). The host ratio in the group with rRNA depletion (0.3–32.2%) was significantly lower (*p* < 0.001, paired *t*-test) than that in the group without rRNA depletion (39.5–90.5%). This is consistent with the results of the whole-genome sequencing of the influenza virus (Figure 3A). The 10× genomic coverage (98.1–99.7%) and target reads per million in the rRNA depletion group were significantly higher (*p* < 0.01, paired *t*-test) than those in the group without rRNA depletion (25.9–99.4%) (Figure 3B,C). Although the correlation between the Ct value of SARS-CoV-2 and genomic coverage reached a significant level, the correlation coefficient Radj2 was only 0.4091 (sample Ct range: 19.5–29.2), which was lower than the correlation coefficient of influenza virus. These results further indicate that for RNA viruses (such as influenza virus and SARS-CoV-2), depletion of rRNA can reduce the host sequences and increase the genomic coverage and proportion of valid data.

### 3.2. Impact of rRNA Probe Capture Degradation on Library Construction Performance

**Figure 4:**
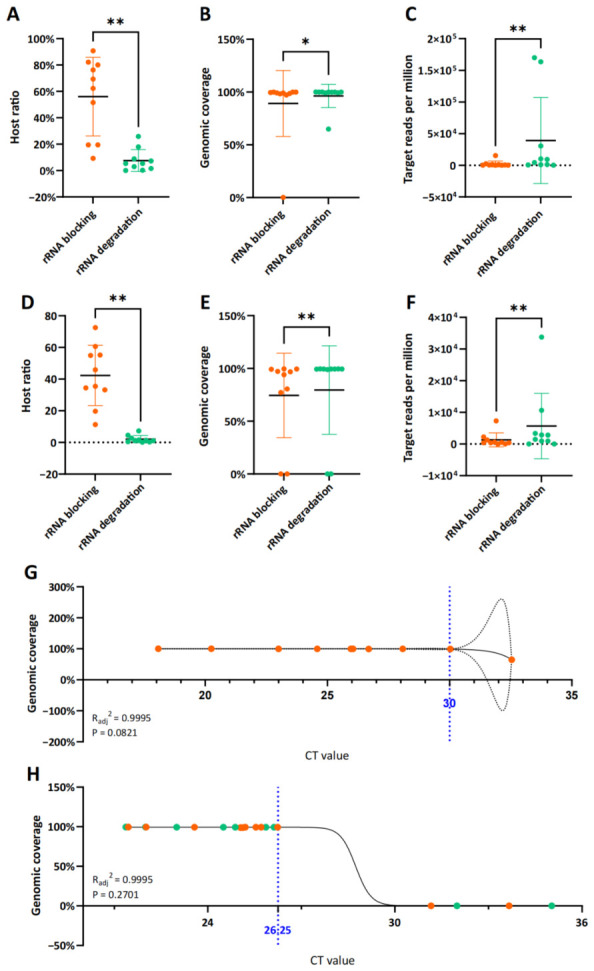
Comparison of SARS-CoV-2 and influenza virus metagenomic sequencing data using rRNA blocking and rRNA degradation. (**A**–**C**) Host ratio, genomic coverage and target reads per million of the two rRNA depletion methods for influenza virus metagenomic sequencing data. (**D**–**F**) Host ratio, genomic coverage and target reads per million of the two rRNA depletion methods for SARS-CoV-2 metagenomic sequencing data. (**G**) Fitted graphs of Ct values and genomic cov-erage of the rRNA degradation samples for influenza virus. The blue dot line means the whole genome could be covered (10× coverage ≥ 99%) with Ct value < 30. (**H**) Fitted graphs of Ct values and genomic coverage of the SARS-CoV-2 samples. Color: orange circle = rRNA blocking samples, green circle = rRNA degradation samples. The blue dot line means the whole genome could be covered (10× coverage ≥ 99%) with Ct value < 26.3. *, *p* < 0.05; **, *p* < 0.01.

After confirming that rRNA depletion significantly reduced the host ratio and increased the proportion of valid data and genomic coverage, we explored the impact of rRNA depletion methods on library construction. We compared the probe capture digestion and rRNA blocking module methods using 10 samples each of the SARS-CoV-2 and influenza virus RNA data. The host ratio (average 8.7%) of SARS-CoV-2 and influenza virus in the rRNA degradation group was significantly (*p* < 0.01, paired *t*-test) lower than that (average 45.3%) in the rRNA blocking group (Figure 4A,D). The rRNA degradation groups for SARS-CoV-2 and the influenza virus consistently showed a significantly (*p* < 0.01, paired *t*-test) higher 10x genomic coverage (average 83.9%) (Figure 4B,E). The target reads per million in the probe-mediated rRNA degradation group were also 1–2 orders of magnitude higher than those in the rRNA blocking group (Figure 4C,F). These results indicate that rRNA degradation is more suitable for library construction for SARS-CoV-2 and influenza virus.

In the fitting analysis of Ct values and genomic coverage for the SARS-CoV-2 and influenza virus, the corrected R2 was close to 1 (Figure 4G,H), unlike the result regarding whether rRNA was depleted, although the statistical test of the correlation fitting was not significant. This indicates a strong correlation between Ct values and viral genomic coverage in the probe-mediated rRNA degradation groups for both the SARS-CoV-2 and influenza virus. The influenza virus samples covered the entire genome well (≥99.5%) when Ct was <30, while the SARS-CoV-2 samples covered the entire genome well (≥99.0%) only when Ct was <26.25. These findings demonstrate that influenza virus exhibits better genomic coverage performance than SARS-CoV-2 at equivalent viral loads.

### 3.3. Comparison of Effects of the Two Fragmenting Methods on Library Construction

**Figure 5:**
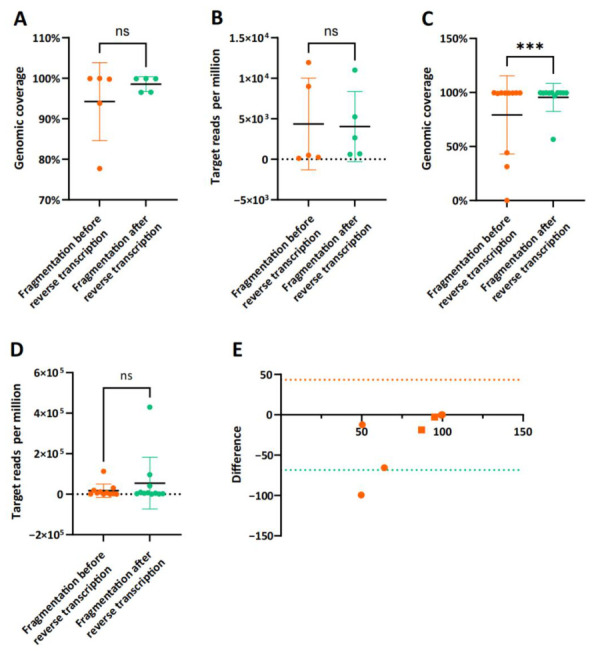
Comparison of SARS-CoV-2 and influenza virus metagenomic sequencing data using fragmentation before reverse transcription and fragmentation after reverse transcription. (**A**,**B**) Genomic coverage and target reads per million of the two strategies for influenza virus metagenomic sequencing data. (**C**,**D**) Genomic coverage and target reads per million of the two strategies for SARS-CoV-2 metagenomic sequencing data. (**E**) Consistency analysis graph (Bland–Altman) of genome coverage for two different library construction strategies (left to right). The orange dots represent the samples using fragmentation before reverse transcription. The square dots represent the influenza virus samples, and the circular dots represent the SARS-CoV-2 samples. The orange/green dotted lines represent the 95% confidence interval. (**E**) Bland–Altman plot for genomic coverage consistency of two fragmentation workflows. X-axis: mean of paired coverage values; Y-axis: coverage difference (fragmentation before reverse transcription minus fragmentation after reverse transcription). Dotted lines denote 95% limits of agreement. Color: orange = fragmentation before reverse transcription; green = fragmentation after reverse transcription. Shape: squares = influenza A; circles = SARS-CoV-2. ns, not significant; ***, *p* < 0.001.

Building on the probe-mediated rRNA degradation method, we further investigated how the timing of reverse transcription and fragmentation affects library construction performance for SARS-CoV-2 and influenza virus samples. There was no significant difference in the target reads per million obtained from 15 samples each of the SARS-CoV-2 and influenza virus using the library construction method of fragmentation after reverse transcription compared with fragmentation before reverse transcription (Figure 5B,D). However, the 10× genomic coverage of the two viruses did not show consistency. There was no significant difference in 10× genomic coverage between the two library construction methods for the influenza virus (fragmentation before reverse transcription: 77.7–100.0% vs. fragmentation after reverse transcription: 96.5–99.9%) (*p* = 0.326). Reverse transcription before fragmentation for library construction for SARS-CoV-2 achieved significantly higher 10× genomic coverage than fragmentation before reverse transcription (*p* < 0.001, paired *t*-test) (Figure 5A,C). We used the Bland–Altman method to analyze the consistency of the 10× genome coverage data obtained using the two library construction methods (Figure 5E). Only the samples from fragmentation before reverse transcription are plotted (all < 0), indicating that the 10× genome coverage obtained by fragmentation before reverse transcription was lower than for reverse transcription before fragmentation. Two SARS-CoV-2 samples (samples 28 and 38) fell outside the 95% limits of agreement, suggesting that these two samples were major contributors to the significantly lower 10× genomic coverage of the fragmentation before reverse transcription for library construction compared to reverse transcription before fragmentation (Figure 5E).

## 4. Discussion

### 4.1. rRNA Depletion Is Key to Improving RNA Virus Metagenomic Library Construction

This study confirmed that rRNA depletion significantly reduced host sequence contamination (by an average of 60–80%) and enhanced the efficiency of capturing viral sequences, especially for samples with higher Ct values (30–32 and low viral load). After rRNA depletion, the coverage increased from <50% to >99%, which is directly related to the high abundance of rRNA in total RNA samples (80–90%). This result is consistent with James et al. (2019) [18]. Depletion of rRNA can reduce the consumption of sequencing resources by host sequences [18,19]. Cerón et al. (2023) revealed that rRNA depletion significantly enhanced the coverage of low viral load samples (Ct values reaching 35). For the samples that originally had a coverage < 85% (11 of 15), their genomic coverage increased from 29.3% to 83.3% and 73.3%, respectively. After rRNA depletion, the interference from human and bacterial rRNAs was reduced, increasing the proportion of the target virus sequence and achieving full coverage, successfully identifying the SARS-CoV-2 genotype [20]. However, our study clarified the optimal application scenarios for clinical samples (such as influenza virus and SARS-CoV-2) (Ct ≤ 32). For clinical samples with low viral loads, such as those collected in the early or recovery stages of infection, rRNA depletion is a necessary step to obtain high-quality whole-genome sequencing data. It increases the proportion of target viral sequences, reduces rRNA background interference, and thereby prevents the failure to detect variant sites caused by incomplete genomic coverage [21].

The correlation between the SARS-CoV-2 genomic coverage and Ct value is weaker than that observed for the influenza virus. This discrepancy is partially attributable to differences in genomic structure and RNA stability between the two viruses. As a single-stranded, positive-sense, non-segmented RNA virus, the 29.7 kb genome of SARS-CoV-2 is large and prone to forming complex secondary/tertiary structures [22]. The viral genome may also be associated with viral proteins or host factors in host cells to form ribonucleoprotein complexes [21], which renders certain genomic regions difficult to amplify efficiently during library preparation, reduces the uniformity of genomic coverage, and weakens the linear correlation with Ct value. In contrast, the segmented genome of influenza virus (13.5–15.5 kb) is smaller and has a simpler structure [23]. Each segment has a more balanced independent amplification efficiency [24], so the correlation between Ct value (reflecting total amount of viral nucleic acid) and genome coverage is higher. Another possible reason is the differences in the virus life cycle and nucleic acid heterogeneity. When SARS-CoV-2 replicates in host cells, it produces abundant subgenomic RNA and incomplete viral nucleic acid fragments [25]. These non-full-length RNAs are captured by the target sequences of the nucleic acid detection kits, resulting in a lower Ct value (suggesting a high viral load). However, the proportion of complete genomes that can be used for genome sequencing is low, thereby reducing the correlation between Ct value and genomic coverage [26]. In the replication of influenza virus, the proportion of subgenomic RNA is lower, and the viral nucleic acid is mainly in the form of complete segments [27]. The Ct value can more accurately reflect the quantity of the complete genome [28].

### 4.2. Probe Capture Degradation Is a Superior Strategy for rRNA Depletion

Despite extensive research on rRNA depletion technologies, direct head-to-head comparisons of different strategies specifically optimized for clinical viral metagenomics remain limited. Recent cross-site evaluations have shown that most commercially available methods can reduce rRNA content to <20% of total reads in high-quality RNA samples [29]. However, these authors also reported that RNase H methods were less variable than pull-out approaches and that some length bias was evident when comparing DGE (differential gene expression) across the different kits. The comparison also described another method, similar to RNase H, that performed well but had not previously been reported. The ZapR method (Takara Bio Inc., Kusatsu, Japan) is a proprietary technology that enzymatically degrades RNA-seq library fragments derived from rRNAs. One limitation of rRNA depletion approaches is that they require a higher read depth per sample than does oligo-dT RNA-seq [30], primarily because of the carry-over of rRNAs.

In the present study, probe-mediated rRNA degradation achieved a higher host depletion efficiency (average host proportion 8.7%) after enzymatic digestion following specific probe binding to rRNA. In contrast, the rRNA blocking module, which relies on competitive binding with the probe, resulted in residual host sequences (average 45.3%) due to insufficient binding efficiency, thereby affecting the coverage of the viral sequence [31]. Everaert et al. detailed the application of the probe competitive binding mechanism in rRNA blocking. Efficient blocking depends on the high-affinity oligonucleotide specifically hybridizing with rRNA. If the probe binding efficiency is insufficient, non-target sequences (including host DNA and unbound rRNA) remain, and optimizing probe design and hybridization conditions can improve the binding efficiency and reduce the residuals [32]. Probe capture directly removes rRNA without merely inhibiting its amplification, making it more suitable for the unbiased capture requirements of RNA virus metagenomics [7]. The blocking module may be affected by factors such as a high rRNA concentration in the sample and insufficient probe affinity [33]. Although probe-mediated RNase H degradation requires an additional 30 min enzymatic step, its superior and more consistent depletion efficiency makes it the preferred strategy for most clinical respiratory RNA virus metagenomics applications. The choice between this method and probe-based rRNA blocking ultimately depends on balancing analytical performance with operational requirements. Probe-mediated RNase H degradation achieves significantly higher and more reproducible host depletion (average host sequence proportion 8.7% vs. 45.3% in our study) with lower susceptibility to sample matrix effects, making it indispensable for low-viral-load samples (Ct ≥ 22, especially 22–32) requiring full-genome assembly for variant calling, outbreak surveillance, or public health investigations. In contrast, probe-based rRNA blocking offers a simpler workflow with fewer pipetting steps, 15–20 min faster turnaround time, and lower reagent costs, making it suitable for high-viral-load samples (Ct < 22) where residual host contamination has minimal impact, large-scale batch screening prioritizing speed, or preliminary qualitative detection assays. For all samples, a post-depletion host sequence proportion of <20% should be used as the key quality control threshold to ensure reliable results [29].

Based on our findings, we strongly recommend mandatory rRNA depletion for all respiratory samples with RT-PCR Ct values ≤ 32 when performing metagenomic sequencing for influenza A virus and SARS-CoV-2. For these samples, probe-mediated RNase H degradation (e.g., Baiyi BK-RLA024) should be used as the first-line method, as it consistently achieves > 90% host rRNA removal and enables ≥ 99% 10× genome coverage in samples with Ct ≤ 31.3 (influenza A virus) and Ct ≤ 26.25 (SARS-CoV-2). For samples with Ct values > 32, rRNA depletion remains beneficial but should be combined with increased sequencing depth (≥200× per sample) [20] to improve genome coverage. Clinical laboratories should monitor the post-depletion host sequence proportion as a key quality control metric, with values < 20% indicating acceptable depletion efficiency for reliable viral detection and genome assembly.

### 4.3. The Fragmentation Sequence Can Be Flexibly Selected According to the Experimental Requirements

Our study demonstrated that there was no significant difference in the library construction quality between the two fragmentation strategies [34,35]. This can be attributed to the high compatibility between reverse transcriptase and fragmentation reagents, as well as the comparable impact of both strategies on RNA integrity (coefficient of variation (CV) of library uniformity < 5%). Wery et al. (2013) [36] were the first to systematically compare the RNase III enzyme method and the magnesium ion chemical method for fragmentation. They reported no significant differences in core quality metrics including library yield, mapping rate, and number of detected genes between the two methods. When M-MuLV (Moloney Murine Leukemia Virus) reverse transcriptase was used, the cDNA synthesis efficiency of both fragmentation methods was >92%, indicating a high compatibility between the reverse transcriptase and the fragmentation enzyme. Meanwhile, the CV values of library uniformity for the two methods were 4.2% (enzymatic method) and 4.5% (chemical method), both of which were below 5%, confirming comparable impacts on RNA integrity. Puchta et al. (2020) specifically evaluated the impact of RNA fragmentation methods on library uniformity. They found that the RIN (RNA integrity number) values of the RNA samples treated with the two fragmentation methods changed by <0.5, indicating a small and comparable impact on RNA integrity [37]. The CV values of library homogeneity were both <4.8%, and further analysis showed that there were no significant differences in indicators such as gene coverage uniformity, GC bias, and end bias between the two methods, confirming that the library construction quality was comparable. Thus, when the integrity of the sample RNA is high (RIN ≥ 7), the option of fragmentation followed by reverse transcription can be chosen, which is operationally more convenient [36]. Conversely, when the sample RNA is severely degraded (RIN < 6), it is recommended to use reverse transcription followed by fragmentation to avoid further degradation of the RNA fragments.

### 4.4. Research Limitations and Future Directions

Although the results of this study were highly consistent with expectations, the study only covered the influenza virus and SARS-CoV-2. Whether the optimized workflow can be applied to other RNA viruses (such as respiratory syncytial virus and adenovirus) remains to be verified. Furthermore, this study only assessed performance in human clinical samples and did not compare rRNA depletion efficiency across other host species. In future work, we will expand the panel of viruses and host species, optimize the rRNA depletion probe panel, and develop an integrated kit combining rRNA depletion, fragmentation, and library construction. This will shorten the experimental period and combine with single-cell sequencing technology to improve the detection sensitivity of low-load clinical samples.

## 5. Conclusions

This study systematically compared key experimental parameters of RNA virus metagenomic library construction and confirmed that rRNA depletion markedly reduces host sequence background noise and improves viral genomic coverage, representing an essential step in library construction for clinical samples. The rRNA depletion efficiency of probe-mediated RNase H digestion is superior to that of the rRNA blocking method, making it the optimal rRNA depletion strategy for clinical viral metagenomic sequencing. The two fragmentation timing strategies (fragmentation before vs. after reverse transcription) exhibit comparable performance and can be flexibly selected according to the RNA integrity status of the samples. This study provides a reliable and practical optimized workflow for RNA virus metagenomic library construction, which supports the standardization of RNA virus identification protocols for pathogens including SARS-CoV-2 and influenza virus and enables rapid pathogen identification and source tracing for infectious disease outbreaks by medical and public health agencies.

## Data Availability

The data presented in this study are openly available in GenBank under BioProject at [https://www.ncbi.nlm.nih.gov/bioproject/?term=PRJNA1400115], 28 June 2026, [PRJNA1400115].
